# SWELLMAP 2, a phyB-Interacting Splicing Factor, Negatively Regulates Seedling Photomorphogenesis in *Arabidopsis*

**DOI:** 10.3389/fpls.2022.836519

**Published:** 2022-02-10

**Authors:** Tingting Yan, Yueqin Heng, Wenwei Wang, Jian Li, Xing Wang Deng

**Affiliations:** ^1^Harbin Institute of Technology, Harbin, China; ^2^Key Laboratory of Molecular Design for Plant Cell Factory of Guangdong Higher Education Institutes, Institute of Plant and Food Sciences, Department of Biology, School of Life Sciences, Southern University of Science and Technology, Shenzhen, China; ^3^State Key Laboratory of Protein and Plant Gene Research, School of Advanced Agricultural Sciences and School of Life Sciences, Peking-Tsinghua Center for Life Sciences, Peking University, Beijing, China

**Keywords:** light signaling, photomorphogenesis, phytochrome B, alternative pre-mRNA splicing, splicing factor

## Abstract

Light-triggered transcriptome reprogramming is critical for promoting photomorphogenesis in *Arabidopsis* seedlings. Nonetheless, recent studies have shed light on the importance of alternative pre-mRNA splicing (AS) in photomorphogenesis. The splicing factors splicing factor for phytochrome signaling (SFPS) and reduced red-light responses in cry1cry2 background1 (RRC1) are involved in the phytochrome B (phyB) signaling pathway and promote photomorphogenesis by controlling pre-mRNA splicing of light- and clock-related genes. However, splicing factors that serve as repressors in phyB signaling pathway remain unreported. Here, we report that the splicing factor SWELLMAP 2 (SMP2) suppresses photomorphogenesis in the light. SMP2 physically interacts with phyB and colocalizes with phyB in photobodies after light exposure. Genetic analyses show that SMP2 antagonizes phyB signaling to promote hypocotyl elongation in the light. The homologs of SMP2 in yeast and human belong to second-step splicing factors required for proper selection of the 3' splice site (3'SS) of an intron. Notably, SMP2 reduces the abundance of the functional *REVEILLE 8 a* (*RVE8*a) form, probably by determining the 3'SS, and thereby inhibits RVE8-mediated transcriptional activation of clock genes containing evening elements (EE). Finally, SMP2-mediated reduction of functional RVE8 isoform promotes phytochrome interacting factor 4 (PIF4) expression to fine-tune hypocotyl elongation in the light. Taken together, our data unveil a phyB-interacting splicing factor that negatively regulates photomorphogenesis, providing additional information for further mechanistic investigations regarding phyB-controlled AS of light- and clock-related genes.

## Introduction

As one of the most essential environmental factors for plants, light modulates various developmental processes of plants throughout their life cycles. Seedlings undergo skotomorphogenesis when grown in darkness, which is characterized by elongated hypocotyls, closed cotyledons and curved apical hooks. While grown in the light, seedlings possess short hypocotyls, expanded cotyledons and developed chloroplasts. This seedling photomorphogenesis process is vital for the survival and autotrophic growth of plants. Plants have evolved multiple photoreceptors to perceive different wavelength of sunlight (Galvão and Fankhauser, 2015; Paik and Huq, 2019). Among these, phytochromes (phyA-phyE in *Arabidopsis*) are responsible for the perception of red (R) and far-red (FR) light. Phytochromes harbor two functional modules: the N-terminal photosensory module (PSM) which perceives light through the chromophore, and the C-terminal output module (OPM) responsible for dimerization, nuclear localization as well as signal transduction (Cheng et al., 2021). Phytochromes exist as two photo-convertible forms, the active Pfr form absorbing FR light and the inactive Pr form absorbing R light (Burgie and Vierstra, 2014; Burgie et al., 2014). After exposure to R light or high R/FR ratio light conditions, the phytochromes (Pr form) will rapidly convert to Pfr form and translocate from cytosol into the nucleus, after which nuclear speckles called photobodies will appear (Klose et al., 2015; Cheng et al., 2021). The size and number of photobodies tightly correlate with the activity of phytochromes (Huq et al., 2003; Chen and Chory, 2011; Klose et al., 2015). In the past decades, numerous case studies have been reported for signaling transduction from phytochromes to transcriptional factors. Photo-activated phytochromes globally regulate transcriptome reprogramming by modulating stability or activity of many key transcription factors, such as PHYTOCHROME-INTERACTING FACTORS (PIFs) and ELONGATED HYPOCOTYL 5 / HY5-HOMOLOG (HY5/HYH) (Al-Sady et al., 2006; Shen et al., 2008; Legris et al., 2019; Cheng et al., 2021). Interestingly, recent evidences have shown that phytochromes also regulate alternative pre-mRNA splicing in response to red light (Shikata et al., 2014; Xin et al., 2017, 2019; Dong et al., 2020).

In eukaryotes, intron-containing pre-mRNA need to undergo a splicing process which removes the introns and joins the flanking exons together to make a mature mRNA. A large dynamic ribonucleoprotein complex called spliceosome accomplish the splicing process through recognizing four loosely conserved nucleotide sequences (Lorković et al., 2000; Wahl et al., 2009; Kornblihtt et al., 2013; Shi, 2017). They are the 5' splice site (5'SS) with a conserved GU nucleotides, the 3'SS with a conserved AG, a branch point (BP) with a conserved adenosine residue and a polypyrimidine tract upstream of the 3'SS (Lorković et al., 2000; Kornblihtt et al., 2013). The major core spliceosome complex is composed of five uridine-rich (U-rich) small nuclear ribonucleoproteins (snRNPs), including U1, U2, U4, U5, and U6 (Wahl et al., 2009; Lee and Rio, 2015; Shi, 2017). Mandatory inclusion of exons and exclusion of introns in mRNA is termed constitutive splicing (CS). By contrast, alternative splicing (AS) events involve the selective inclusion of introns or exons from pre-mRNA into mature mRNA, and different mRNA isoforms from a single gene will be produced. There are four major types of AS events in plants, including exon skipping (ES), intron retention (IR), alternative 5' splice site (A5'SS) and alternative 3' splice site (A3'SS) (Marquez et al., 2012; Reddy et al., 2013). The subtle regulation of AS is rather complicated. Splicing factors (SFs), such as heterogeneous nuclear ribonucleoproteins (hnRNPs) and serine–arginine repeat proteins (SRs), are essential for regulating AS through binding to cis-regulatory elements (silencers or enhancers) (Kornblihtt et al., 2013; Lee and Rio, 2015). The interaction between SFs and snRNPs can also change the splice sites determination in pre-mRNA (Kornblihtt et al., 2013; Lee and Rio, 2015). In addition, the chromatin-based effects, such as recruitment of SFs by “adaptors” of histone modifications and transcriptional elongation rate, also play roles in changing the splicing patterns (Kornblihtt et al., 2013; Lee and Rio, 2015).

Genome-wide analyses have revealed that a large scale of AS profiles changed in response to light in *Arabidopsis thaliana* (Shikata et al., 2014; Hartmann et al., 2016). Gene ontology (GO) analyses show that numerous genes regulated by light-controlled AS are involved in “response to light stimulus,” “circadian clock” and “photosynthesis” biological processes (Shikata et al., 2014; Xin et al., 2017, 2019). To better understand the molecular mechanism by which light regulates these AS patterns, numerous studies have focused on this in the past few years. It is reported that light-triggered nuclear AS is regulated by a chloroplast retrograde signal (Petrillo et al., 2014). Moreover, light-increased transcriptional elongation rate modulates AS decisions (Godoy Herz et al., 2019). Recently, it is shown that photosynthesized sugars, a shoot-to-root mobile signal, coordinate AS responses to light throughout the whole plant in a TOR kinase-dependent manner (Riegler et al., 2021). Phytochromes are the best studied photoreceptors involved in the regulation of red light-mediated AS till date (Shikata et al., 2012, 2014; Wu et al., 2014; Shih et al., 2019; Lin et al., 2020; Kathare and Huq, 2021). Photo-activated phyB induces a specific intron retention in 5' UTR of *PIF3* mRNA, thereby inhibits PIF3 protein synthesis to promote photomorphogenesis (Dong et al., 2020). REDUCED RED-LIGHT RESPONSES IN CRY1CRY2 BACKGROUND1 (RRC1) and SPLICING FACTOR FOR PHYTOCHROME SIGNALING (SFPS) are identified as the two splicing factors which can directly interact with phyB in *Arabidopsis* (Xin et al., 2017, 2019). These two SFs can form a complex and coordinate pre-mRNA splicing of a subset of light- and clock-associated genes to promote photomorphogenesis (Xin et al., 2017, 2019). For instance, RRC1/SFPS can directly associate with clock regulator EARLY FLOWERING 3 (ELF3) pre-mRNA to regulate its proper splicing. Moreover, PIFs are reported to act downstream of RRC1/SFPS in the regulation of photomorphogenesis (Xin et al., 2017, 2019).

Here we identify another splicing factor SWELLMAP 2 (SMP2) that can physically interact with phyB. SMP2 genetically acts downstream of phyB, and promotes A3'SS of key clock regulator *RVE8* to negatively regulate seedling photomorphogenesis in *Arabidopsis*.

## Materials and Methods

### Plant Materials and Growth Conditions

The ecotype of *Arabidopsis thaliana* used in this study was Columbia-0 (Col-0). T-DNA insertion mutant *smp2-1* (Salk_022202) was from Arabidopsis Biological Resource Center (ABRC), *smp2-3* mutant was generated by CRISPR/Cas9 (Wang et al., 2015). *phyB-9* mutant, *phyB-CFP* and *PIF4-ox* transgenic plants were reported previously (Reed et al., 1993; Chen et al., 2005; Lee et al., 2020). Seeds were sterilized with 20% (v/v) bleach containing 0.1% Triton X-100 for 10 min, washed at least five times with sterile water, and sown on 1 × Murashige and Skoog (MS) medium supplemented with 1% sucrose and 0.8% agar. After 3 days of stratification at 4°C in darkness, seeds were transferred into the plant growth chamber (PERCIVAL) maintained at 22°C.

### Measurement of Hypocotyl Length

To measure the hypocotyl length of seedlings, seeds were sown on plates and stratified in darkness at 4°C for 3 d, followed by incubation in continuous white light for 12 h to induce synchronous germination. The plates were then transferred to continuous dark (D), white (W), red (R), far-red (FR), and blue (B) light conditions and incubated at 22°C for 5 days and the hypocotyl length of seedlings were measured by ImageJ software.

### Plasmid Construction

To generate pLexA-*phyB* constructs for yeast two-hybrid assay, N-terminal (1-651 aa) and C-terminal (652-1172 aa) fragments of *phyB* CDS were amplified by Super-Fidelity DNA Polymerase (Vazyme) and inserted into the *Eco*R I/*Xho* I sites of pLexA vector (Clontech). For pB42AD-*SMP2* plasmid, full-length *SMP2* CDS was inserted into *Eco*R I/*Xho* I sites of pB42AD (Clontech).

For firefly luciferase complementation imaging (LCI) assays, the full-length *phyB* CDS were inserted into the *Kpn* I*/Sal* I sites of pCambia1300-*nLUC*, and *SMP2* CDS were inserted into the *Bam*H I*/Sal* I sites of pCambia1300*-cLUC*.

To generate overexpression of *YFP-SMP2* construct, the Gateway cloning technology was used. Full-length *SMP2* open reading frame was cloned into the *pDONR-223* vector using Gateway BP Clonase Enzyme mix (Invitrogen), and introduced into the plant binary vector *pEarley Gateway 104* under the control of the 35S promoter using Gateway LR Clonase Enzyme mix (Invitrogen).

### Generation of Transgenic Plants

The *pEarley Gateway-YFP-SMP2* construct was transformed into *Agrobacterium tumefaciens* GV3101 by the freeze-thaw method and introduced into Col-0 via the floral dip method (Clough and Bent, 1998). Transgenic plants were selected on MS medium containing 20 mg/L Basta.

### Yeast Two-Hybrid Assay

Yeast two hybrid assays in the LexA system were performed according to the Yeast Protocols Handbook (Clontech). Yeast strain EGY48 containing p8op-LacZ plasmid is used in the study. Transformants were first selected and grown on minimal synthetic defined (SD) base supplemented with -His-Trp-Ura dropout at 30°C, and then transferred to SD/-His-Trp-Ura dropout plates containing 80 mg/L X-gal for blue color development.

### LCI Assay

LCI assays were performed as described previously (Chen et al., 2008). The nLUC- and cLUC-fused plasmids were transformed into *Agrobacterium* strain GV3101, and the indicated transformants were mixed and infiltrated into *Nicotiana benthamiana* leaves. The plants were grown in darkness for 2 d followed by 24h red light exposure. After that, the luciferase signals were measured using NightShade LB985 (Berthold Technologies). The experiments were performed with three biological replicates.

### Co-IP Assay

For Co-IP assay, *YFP-SMP2*, Col-0 and *phyB-9* seedlings were used. The total proteins were extracted by Lysis buffer [50 mM Tris-HCl pH7.5, 150 mM NaCl, 10% glycerol, 0.05% Tween 20, 1 mM PMSF, 1 × protease inhibitor cocktail (Roche)]. Four hundred μg total proteins were incubated with 10 μL GFP-Trap agarose (Chromotek) and rotated at 4°C for 4 h. After incubation, the agarose was washed five times with wash buffer [50 mM Tris-HCl pH7.5, 300 mM NaCl, 10% glycerol, 1 mM PMSF, 1 × protease inhibitor cocktail (Roche)]. The precipitates were boiled in 1 × SDS loading buffer for 10 min, and then the supernatants were analyzed by western blot using anti-GFP and anti-phyB antibodies, respectively.

### Immunoblot Analysis and Antibodies

Five-day-old Arabidopsis seedlings were homogenized in a protein denatured extraction buffer (100 mM NaH_2_PO_4_ pH8.0, 10 mM Tris-HCl pH8.0, 200 mM NaCl, 8 M urea, 1 mM PMSF, 1 × protease inhibitor cocktail). Antibodies used in this study were anti-phyB (PhytoAB), anti-GFP (Abmart) and anti-Actin (Sigma-Aldrich).

### Confocal Microscopy

Subcellular localization observation of YFP-SMP2 and PHYB-CFP were performed using a confocal laser scanning microscope Zeiss LSM880 (Carl Zeiss). For YFP fluorescence detection, the excitation wavelength was 514 nm and the emission spectra were collected from 519 to 620 nm. For CFP fluorescence detection, the excitation wavelength was 405 nm and the emission spectra were collected from 410 to 513 nm.

### RNA Isolation and Quantitative Real-Time PCR Analysis

Total RNA was extracted from *Arabidopsis* whole seedlings with indicated treatments using the Plant RNA kit (Omega). First-strand cDNAs were synthesized from 2 μg of total RNA using 5 × All-In-One RT Master Mix (Applied Biological Materials) according to the manufacturer's instructions. Real-time qPCR was performed using QuanStudio^TM^ 6 Flex Real-Time PCR detection system (Applied Biosystems) and Hieff qPCR SYBR Green Master Mix (YEASEN). The expression levels were normalized to that of *PP2A* gene. The primers used in this study were listed in Supplementary Table 1.

## Results

### phyB Physically Interacts With SMP2

**Figure 1:**
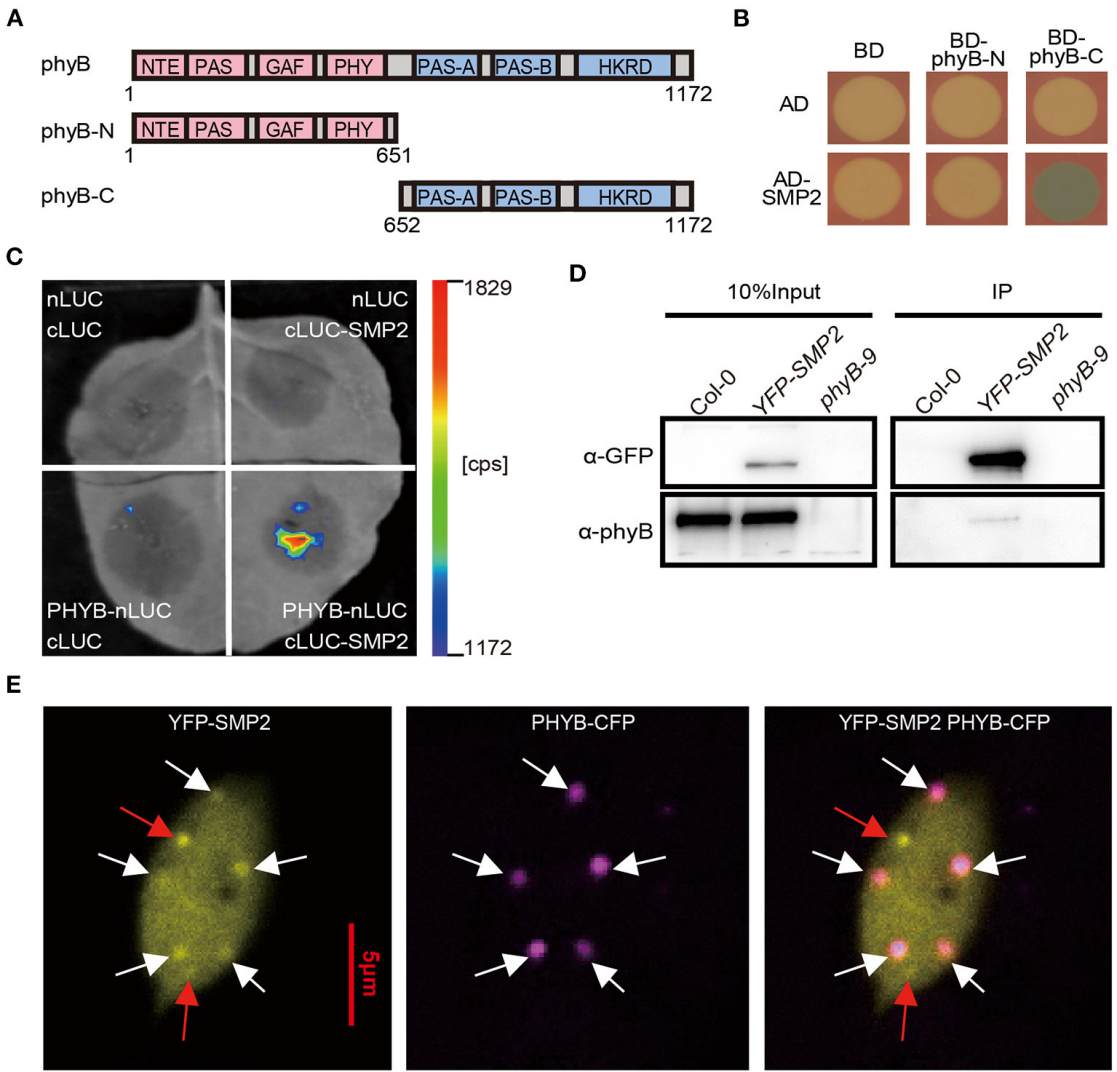
phyB physically interacts with SMP2 *in vitro* and *in vivo*. **(A)** Schematic diagram of phyB fragments. Numbers indicate the amino acid positions in phyB protein. **(B)** The interaction of SMP2 and phyB in yeast. AD, activation domain; BD, DNA-binding domain. **(C)** Firefly luciferase complementation imaging (LCI) assay showing interaction between phyB and SMP2 in tobacco leaf. nLUC, the N-terminal fragment of firefly luciferase (LUC); cLUC, the C-terminal fragment of LUC. Full-length phyB and SMP2 were fused to the nLUC and cLUC, respectively. **(D)** Co-IP assay showing the association between SMP2 and phyB. Seedlings grown in the dark were transferred to red light (145 μmol/m^2^·s) for 1 h. YFP-SMP2 proteins were pulled down with GFP-trap beads. α-GFP, anti-GFP antibody; α-phyB, anti-phyB antibody. **(E)** Colocalization analysis of SMP2 and phyB *in vivo*. Transgenic plants co-expressing *YFP-SMP2* and *phyB-CFP* were grown in continuous red light (145 μmol/m^2^·s) for 5 days. The images of nucleus came from a hypocotyl cell. YFP-SMP2 fusion proteins were excited by laser at 514 nm, and the emitted fluorescence signaling was collected from 519 nm to 620 nm; phyB-CFP were excited by laser at 405 nm, and the emitted fluorescence was collected from 410 nm to 513 nm. Scale bar, 5 μm. White arrowheads indicate SMP2 and phyB colocalized in photobodies; Red arrowheads indicate the SMP2-specific nuclear speckles.

Using the C-terminal output module of phyB as bait, we performed a yeast two-hybrid screen to look for additional splicing regulators involved in light signaling. We found that a protein called SWELLMAP 2 (SMP2), whose homolog Slu7/hSlu7 is a second-step splicing factor required for proper 3' splice site selection (Frank and Guthrie, 1992; Chua and Reed, 1999; Clay and Nelson, 2005), interacted with C-terminal of phyB in yeast (Figures 1A,B). To further verify the interaction between phyB and SMP2 *in vivo*, we first carried out split-luciferase complementation assay. As shown in Figure 1C, an appreciable bioluminescence signal was detected only when phyB-nLUC and cLUC-SMP2 were co-expressed in tobacco leaf. We further performed co-IP assay using transgenic plant overexpressing YFP-SMP2 (Supplementary Figure 1), and the data show that endogenous phyB protein could be co-immunoprecipitated by YFP-SMP2 in red-light condition (Figure 1D), suggesting that phyB was associated with SMP2 *in vivo*. What's more, to investigate whether SMP2 and phyB colocalized with each other *in vivo*, we crossed *YFP-SMP2* with *phyB-CFP* transgenic plants. As shown in Figure 1E, YFP-SMP2 distributed mainly in the nucleus when transgenic plants were grown under continuous red light, and some YFP-SMP2 could colocalize with phyB-CFP in photobodies. Taken together, these results demonstrate that phyB physically interacts with SMP2 *in vitro* and *in vivo*.

### SMP2 Negatively Regulates Seedling Photomorphogenesis

**Figure 2:**
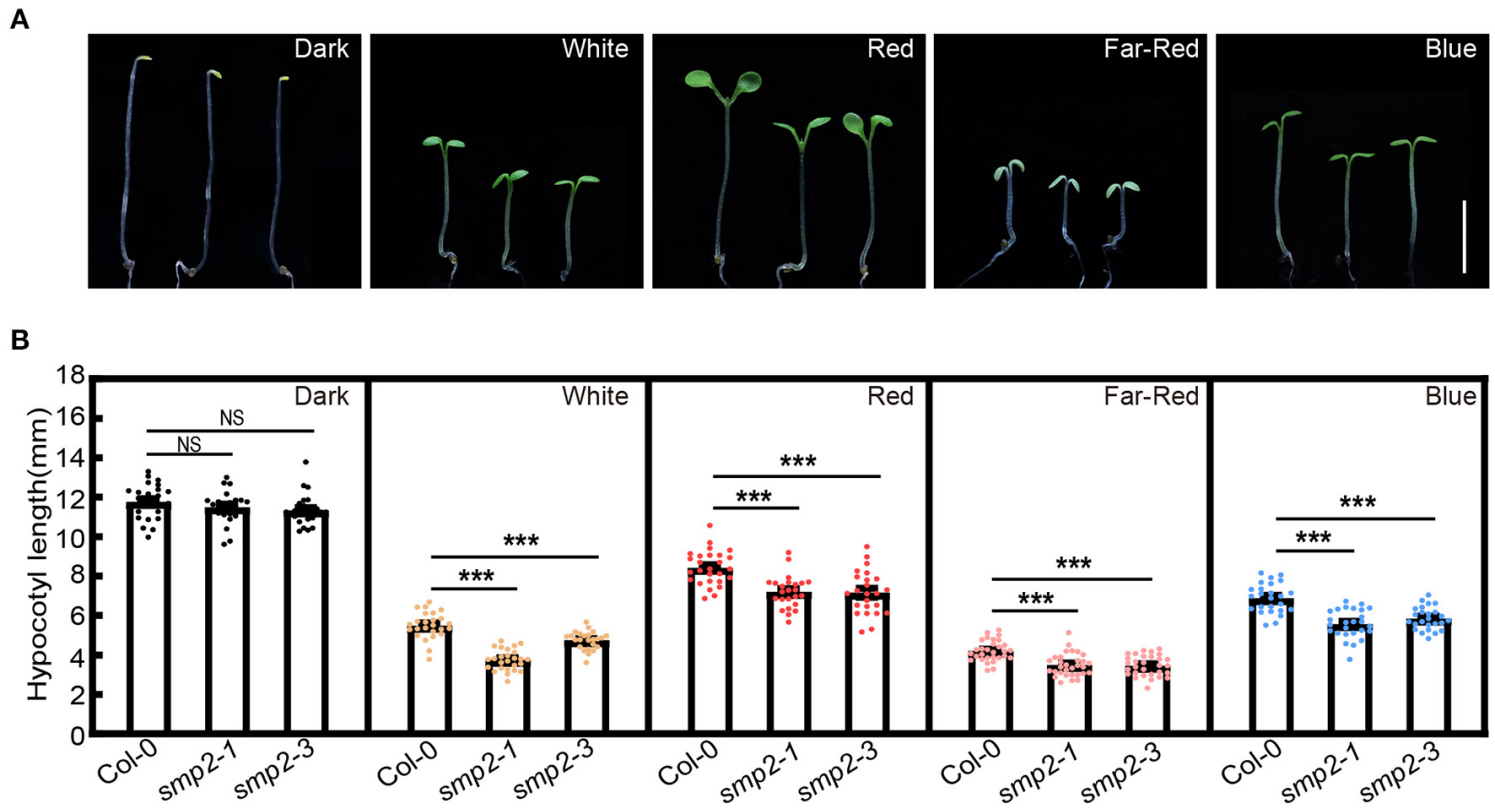
*smp2* mutants are hypersensitive to light. **(A)** Visual phenotypes of 5-day-old wild-type (Col-0) and *smp2* seedlings grown in the dark or different light conditions. White light, 7.5 μmol·m^−2^·s^−1^; Red light, 47 μmol·m^−2^·s^−1^; Far-red light, 4.5 μmol·m^−2^·s^−1^; Blue light, 3.0 μmol·m^−2^·s^−1^. Scale bars, 5 mm. **(B)** Quantification of hypocotyl lengths of Col-0 and *smp2* seedlings grown under conditions as indicated in **(A)**. Error bars represent standard deviation (SD), n ≥ 25; ***P < 0.001 (*t-test*); NS, not significant (*t-test*). Experiments were performed three times with similar results.

To investigate whether SMP2 is involved in light-controlled morphogenesis, a *smp2* null mutant, *smp2-3*, was generated by the Clustered Regulatory Interspaced Short Palindromic Repeats (CRISPR)/Cas9 technique. *Smp2-3* contained a 519-bp deletion within the *SMP2* genomic DNA and could produce a truncated 62-amino-acid protein resulting from a premature stop codon (Supplementary Figure 2). Additionally, the T-DNA insertion mutant *smp2-1* (SALK_022202) (Clay and Nelson, 2005; Liu et al., 2016) was also used in this study. Both *smp2* mutants showed a similar etiolated phenotype as Col-0 (wild-type) when grown in the dark (Figure 2; Supplementary Figure 3). However, both *smp2-1* and *smp2-3* displayed significantly shorter hypocotyls compared to Col-0 when grown in continuous white, red, far-red and blue light conditions (Figure 2; Supplementary Figure 3). These data indicate that SMP2 promotes hypocotyl elongation of seedlings in the light.

### SMP2 Genetically Acts Downstream of phyB to Regulate Hypocotyl Elongation

**Figure 3:**
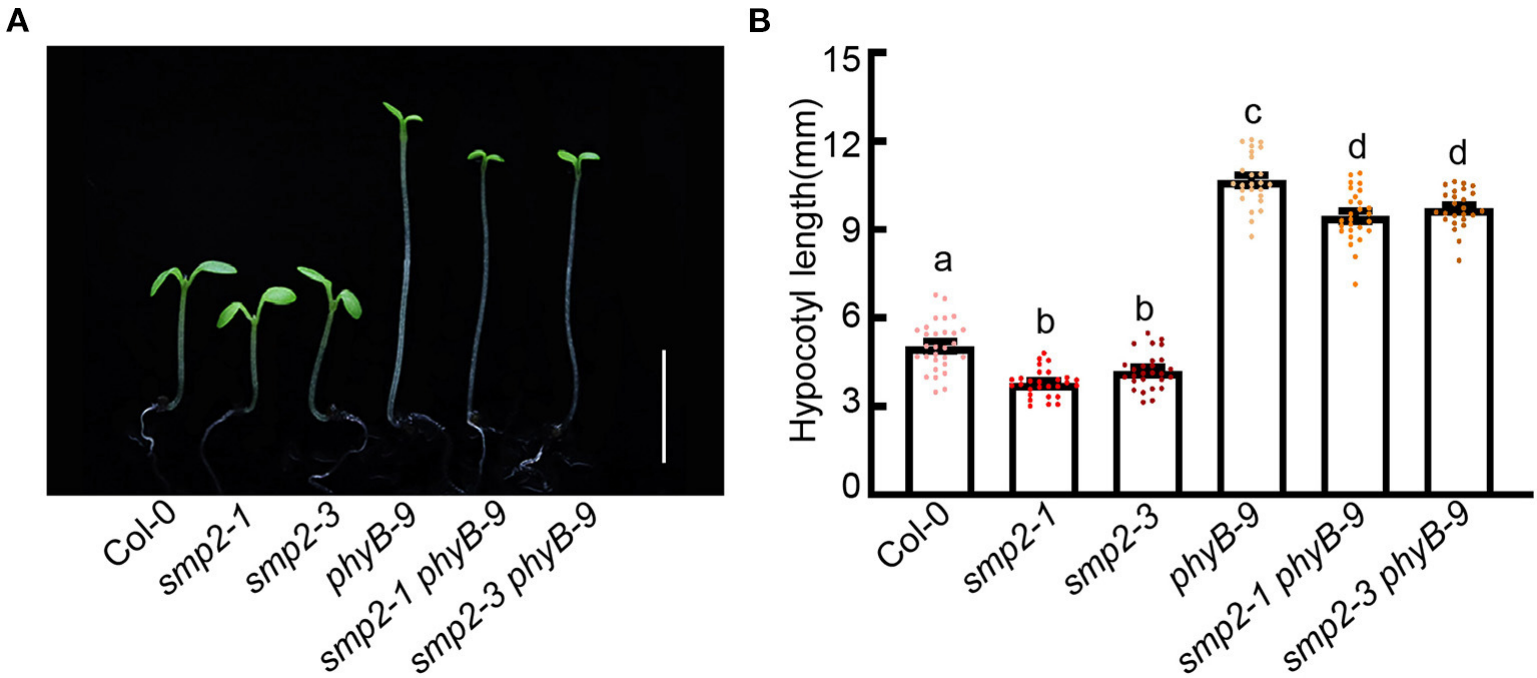
SMP2 genetically acts downstream of phyB. **(A)** Visual phenotypes of wild-type (Col-0), *smp2-1, smp2-3, phyB-9, smp2-1 phyB-9*, and *smp2-3 phyB-9* seedlings grown for 5 days in continuous red-light condition (145 μmol·m^−2^·s^−1^). Scale bars, 5 mm. **(B)** Quantification of hypocotyl length of different seedlings as shown in **(A)**. Error bars represent SD, n ≥ 25. Letters above the bars indicate significant differences (*P* < 0.05), as determined by one-way analysis of variance (ANOVA) with Duncan's *post-hoc* analysis. Experiments were performed three times with similar results.

As shown in Figure 1, the biochemical evidences show that SMP2 physically interacts with phyB. To further explore the genetic relationship between phyB and SMP2, we respectively generated the double mutant *smp2-1 phyB-9* and *smp2-3 phyB-9* through genetic crossing (Supplementary Figure 4). Given that phyB plays prominent role in red light signaling, these mutants were grown in red light and hypocotyl lengths were measured. *phyB-9* exhibited extremely long hypocotyl in red light as previously reported, while both *smp2-1 phyB-9* and *smp2-3 phyB-9* mutants showed significantly shorter hypocotyl compared with *phyB-9* in red light (Figure 3; Supplementary Figure 5). This genetic analysis indicates that SMP2 genetically acts downstream of phyB to promote hypocotyl elongation in red light.

### SMP2 Regulates AS of Circadian Clock Gene *RVE8*

**Figure 4:**
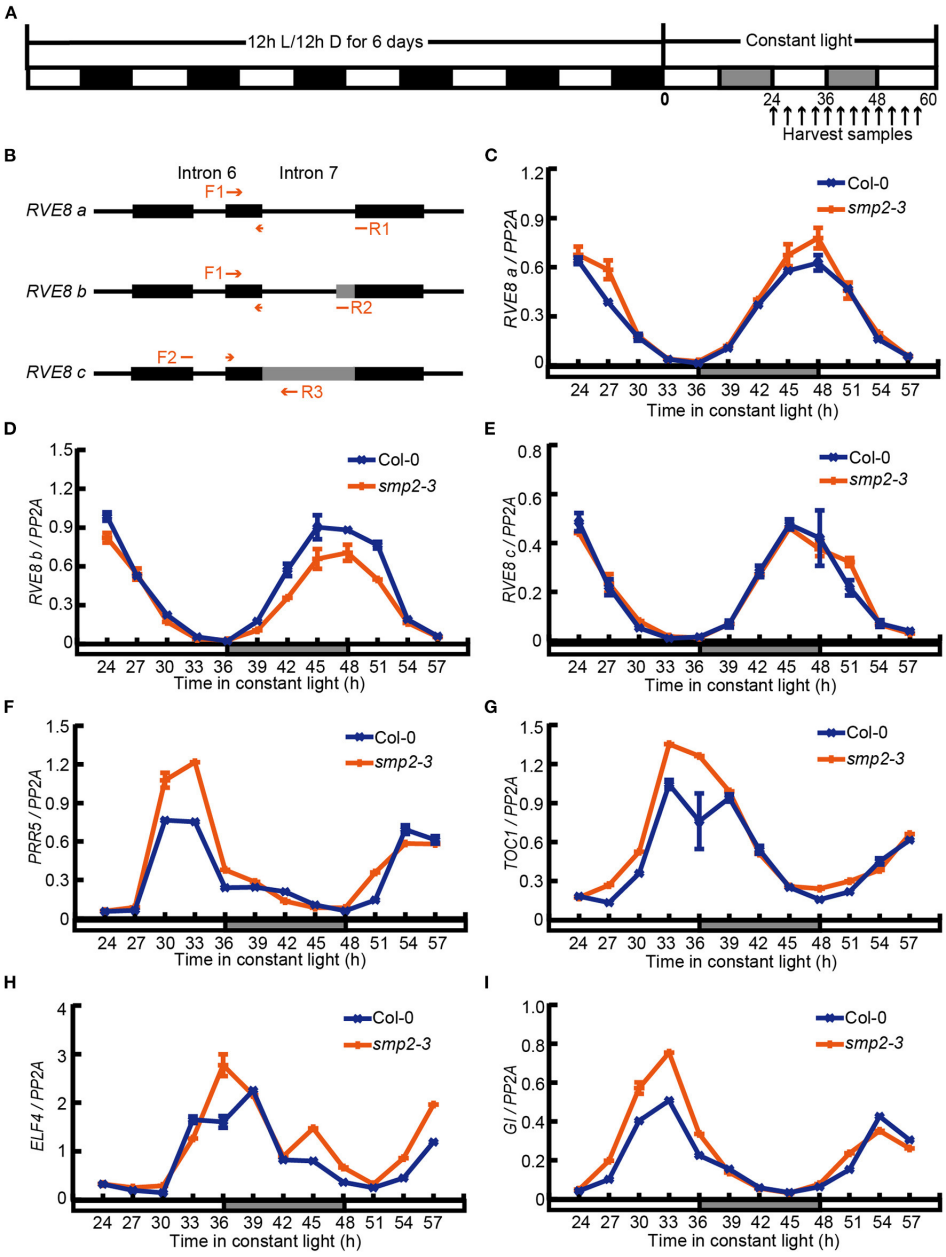
SMP2 regulates A3'SS in *RVE8* and inhibits the expression of RVE8-activated genes. **(A)** Simplified diagram of the diurnal entrainment of seedlings. Plants were entrained in the diurnal cycles (12 h light/12 h dark) for 6 days and then transferred to continuous white light (145 μmol·m^−2^·s^−1^). Seedlings were harvested at the indicated time points. **(B)** The schematic graph of splice variants of *RVE8* in intron 7. Red arrows indicated primers used to amplify specific *RVE8* transcript isoforms. Exons were depicted as black rectangles; introns were depicted as horizontal lines; alternative splicing regions were depicted as gray rectangles. **(C–E)** The relative abundance of three *RVE8* isoforms in Col-0 and *smp2-3*. Total RNA was extracted from Col-0 and *smp2-3* seedlings treated as described in **(A)**. *PP2A* was used as the internal control. **(F–I)** The relative expressions of *PRR5, TOC1, ELF4*, and *GI* in Col-0 and *smp2-3*. Error bars represent SD, *n* = 3. All of these experiments were performed three times with similar results.

Previous transcriptome analyses have revealed that several circadian clock regulators are subject to light-induced alternative splicing (Shikata et al., 2014; Mancini et al., 2016). In addition, phyB-interacting splicing factors, SFPS and RRC1, could also modulate photomorphogenesis by controlling pre-mRNA splicing of light signaling and clock genes such as *CBK1, ELF3*, and *RVE8* (Xin et al., 2017, 2019). To further investigate whether SMP2 was involved in regulating AS of circadian clock regulators, we harvested seedlings released to constant white light (LL) after 6-day diurnal entrainment (Figure 4A) and monitored the AS patterns of various circadian clock genes in Col-0 and *smp2-3* by RT-PCR and qPCR. Interestingly, we found that the AS patterns of *RVE8* (James et al., 2012; Mancini et al., 2016) were altered in *smp2-3* (Supplementary Figure 6). *RVE8a* was generated by the complete exclusion of intron 7, and two extra AS events happened within *RVE8* intron 7 were also validated by sequencing. *RVE8b* was generated by an alternative 3' splice site selection that inserted 22nt into *RVE8* mRNA, whereas *RVE8c* was generated by the complete retention of the intron 7 (Figure 4B). To quantify the abundance of these three isoforms, we designed three primer pairs to amplify specific transcripts (Figure 4B). The results showed that the expression of all three isoforms exhibited rhythmic changes in both Col-0 and *smp2-3* (Figures 4C–E). However, the peak abundance of the functional isoform *RVE8a* was higher in *smp2-3* than in Col-0 (Figure 4C), whereas the isoform *RVE8b* was significantly lower in *smp2-3* than in Col-0 (Figure 4D). In contrast, the abundance of the isoform *RVE8c* was similar in *smp2-3* and Col-0 (Figure 4E). RVE8, which encodes a MYB-like transcription factor, is a homolog of the key clock regulators CCA1 and LHY (Farinas and Mas, 2011; Rawat et al., 2011). The three transcription factors all bind specifically to the Evening Element (EE) promoter motif (Hsu et al., 2013; Shalit-Kaneh et al., 2018). The difference is that RVE8 activates the expression of EE-containing clock genes, while CCA1/LHY represses the expression of these genes (Hsu et al., 2013; Shalit-Kaneh et al., 2018). To test whether increasing *RVE8a* expression led to increased function of RVE8 in *smp2-3*, we quantified the expression of RVE8-activated genes in *smp2-3*. The results showed that consistent with the higher abundance of the functional *RVE8a* isoform in *smp2-3*, these evening-phased genes, including *PRR5, TOC1, ELF4* and *GI*, exhibited increased rhythmic amplitudes in *smp2-3* than in Col-0 (Figures 4F–I). Taken together, these data suggest that SMP2 is involved in promoting A3'SS in *RVE8* intron 7 and disturbing the expression of RVE8-activated genes.

### SMP2-Mediated Hypocotyl Elongation Partially Depends on *PIF4*

**Figure 5:**
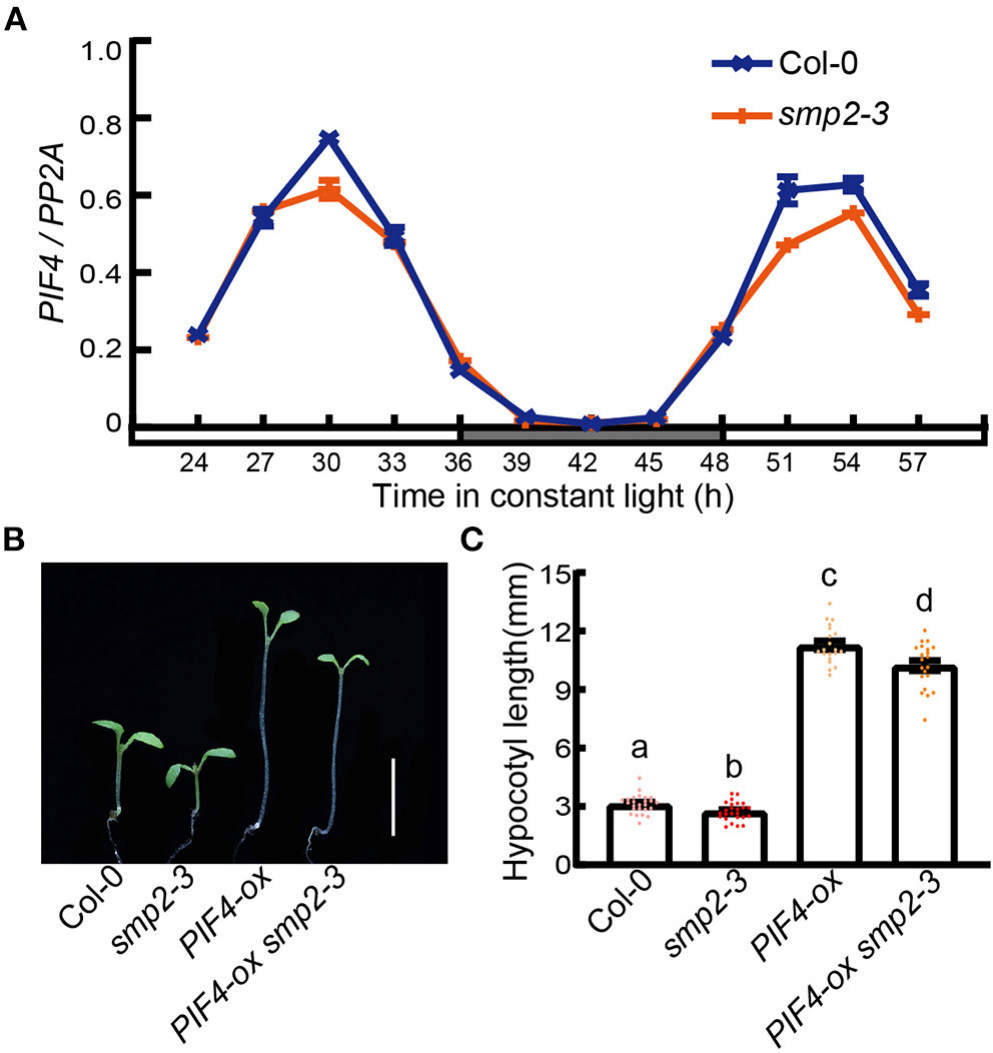
PIF4 contributes to SMP2-mediated hypocotyl elongation. **(A)** The relative expression of *PIF4* in Col-0 and *smp2-3*. Seedlings were entrained in the diurnal cycles (12 h light/12 h dark) for 6 days and then transferred to continuous white light (145 μmol·m^−2^·s^−1^). Seedlings were harvested at the indicated times. The relative expression levels of *PIF4* were normalized to *PP2A*. Error bars represent SD, *n* = 3. **(B)** Visual phenotypes of 5-day-old Col-0, *smp2-3, PIF4-ox* and *PIF4-ox smp2-3* seedlings grown in continuous red light condition (145 μmol·m^−2^·s^−1^). Scale bars, 5 mm. **(C)** Quantification of hypocotyl length of Col-0, *smp2-3, PIF4-ox* and *PIF4-ox smp2-3* seedlings grown in continuous red light. Error bars represent SD, n ≥ 20. Letters above the bars indicate significant differences (*P* < 0.05), as determined by one-way analysis of variance (ANOVA) with Duncan's *post-hoc* analysis.

To investigate whether SMP2 indeed affects the output of circadian clock, we also performed qPCR to examine the expression level of *PIF4*, whose expression exhibited diurnal rhythm with a peak in the subjective afternoon (Figure 5A; Nusinow et al., 2011). However, the peak amplitude of *PIF4* expression was depressed in the *smp2-3* mutant (Figure 5A), which possibly resulted from the higher abundance of *RVE8a* transcript in *smp2-3* (Gray et al., 2017).

To further confirm whether PIF4 was involved in SMP2-mediated hypocotyl elongation, we crossed an over-expression line of *PIF4* (*PIF4-ox*) with *smp2-3* and analyzed their phenotypes. The hypocotyls of *PIF4-ox* seedlings were dramatically longer than those of Col-0 in red light as previously described, indicating the amplification of functional PIF4 proteins in *PIF4-ox* lines. Furthermore, the hypocotyls of seedlings overexpressing *PIF4* in *smp2-3* were significantly longer than those of *smp2-3*, but slightly shorter than those of *PIF4-ox* in red light (Figures 5B,C; Supplementary Figure 7). Together, these results demonstrate that SMP2-mediated hypocotyl elongation in red light partially depends on functional PIF4.

## Discussion

SMP2 and its paralog SMP1 are conserved in evolution, for example, Slu7 and hSlu7 are the homolog of SMP2 in yeast and human, respectively (Frank and Guthrie, 1992; Chua and Reed, 1999). Slu7/hSlu7 is a second-step splicing factor required for proper selection of the 3' splice site (Frank and Guthrie, 1992; Chua and Reed, 1999). The *smp1smp2* double mutant in *Arabidopsis* is not viable, indicating their indispensable roles in plant development (Clay and Nelson, 2005; Liu et al., 2016). Moreover, the interaction between SMP1/2 and SKIP, a component of the spliceosome in *Arabidopsis*, is reported to be conserved in yeast and human (Liu et al., 2016). Similar to the splicing defect in *skip* mutant, knockout of *SMP1/2* in *Arabidopsis* protoplasts leads to significant accumulation of aberrant splicing products of certain genes (Liu et al., 2016), suggesting that SMP1/2 are also involved in pre-mRNA splicing in plants. In this study, we demonstrated that phyB physically and functionally interacted with SMP2 in response to light. SMP2 decreased the abundance of functional *RVE8a*, likely by regulating the 3'SS determination, resulting in a decrease in evening-phased genes expression and a subsequent promotion of *PIF4* expression to fine-tune seedling photomorphogenesis. In addition, *smp2* mutants also showed short hypocotyls under far-red and blue light (Figure 2), suggesting that SMP2 might also be involved in the phyA and cryptochromes signaling pathways to regulate photomorphogenesis.

The key clock regulator *RVE8* gene produces various mRNA isoforms, and our work revealed that SMP2 might be involved in the 3'SS determination of *RVE8* intron 7 (Figure 4). Lack of SMP2 resulted in a decrease in the abundance of *RVE8b* isoform with a concomitant increase in the abundance of the functional *RVE8a* isoform (Figures 4C,D). RT-qPCR confirmed that expression levels of RVE8 target genes were increased in *smp2-3* mutant, suggesting that the higher expression of *RVE8a* was responsible for the increased function of RVE8 in *smp2-3*. However, the mechanism of how the different isoforms of *RVE8* affect RVE8 function remains unclear. In general, AS events can lead to the production of premature termination codons (PTCs) in mRNA, which can subsequently be degraded by nonsense-mediated mRNA decay (NMD) or produce truncated proteins with distinct functions (Seo et al., 2012; Reddy et al., 2013; Chaudhary et al., 2019). In addition, some AS transcripts can be sequestered in the nucleus and spliced as needed to respond to varying environmental conditions (Reddy et al., 2013; Petrillo et al., 2014; Filichkin et al., 2015). Therefore, further investigation of the fates of the different *RVE8* AS isoforms is required to unravel the biological significance of SMP2-mediated AS.

RVE8 can directly activate the expression of genes containing EE-motif in their promoters (Hsu et al., 2013), which leads to mis-regulation of *PIF4* and *PIF5* expression and modulation of seedling photomorphogenesis (Gray et al., 2017). In this study, we verified the *PIF4* expression is lower in *smp2-3* than in Col-0, and overexpression of *PIF4* in *smp2-3* can restore the short hypocotyl of *smp2-3* in the light (Figure 5). These demonstrate that PIF4 contributes to SMP2-mediated hypocotyl elongation in the light. In addition, we also detected the rhythmic *PIF5* expression in *smp2-3* (Supplementary Figure 8). Similar to the *PIF4* expression pattern, *PIF5* also showed reduced peak expression in *smp2-3*, this suggests that PIF5 may also play a role in SMP2-mediated regulation of photomorphogenesis. In this way, the slightly shorter hypocotyl of *PIF4-ox smp2-3* compared to *PIF4-ox* seedlings can be partially explained (Figure 5).

To date, the question of how splicing factors are regulated by light signal remains rather elusive. Previous studies have shown that the transcription level and protein stability of SFPS/RRC1 are not regulated by phyB and light signal (Xin et al., 2017, 2019). However, in moss the splicing regulator PphnRNP-F1 is reported to be stabilized by red light, which depends on PpPHY4 (Lin et al., 2020). In this study, the expression level of *SMP2* was also not regulated by red light (Supplementary Figure 9). Whether the protein stability of SMP2 is regulated by light requires further investigation. The genetic interaction of SMP2 and phyB suggests that functional phyB contributes to the short hypocotyl of *smp2* mutants (Figure 3). The shorter hypocotyls of *smp2-1 phyB-9* and *smp2-3 phyB-9* compared with the *phyB-9* single mutant suggest that the other phytochromes (Cheng et al., 2021) may also be involved in SMP2-mediated regulation of photomorphogenesis in red light. Given that active phytochromes can induce phosphorylation and degradation of PIFs (Legris et al., 2019; Cheng et al., 2021), it is likely that phyB induces the phosphorylation of SMP2 to fine-tune its activity in response to red light. In addition, these phyB-interacting splicing factors, previously reported SFPS/RRC1 and SMP2 in this study, partially co-localized with phyB in nuclear photobodies in prolonged red light condition (Figure 1E) (Xin et al., 2017, 2019). The size and number of photobodies correlate closely with phyB activity (Klose et al., 2015; Legris et al., 2019; Cheng et al., 2021). And the relative concentration of splicing regulators is crucial for splice site selection during spliceosome assembly (Shomron et al., 2005; Saltzman et al., 2011; Kornblihtt et al., 2013). These facts provide another hypothesis that activated phyB may regulate the subnuclear localization of SMP2 and modulate its functions in alternative pre-mRNA splicing of certain light- and clock-associated genes.

## Data Availability Statement

The original contributions presented in the study are included in the article/Supplementary Material, further inquiries can be directed to the corresponding authors.

## Author Contributions

TY, JL, and XD designed the research and wrote the paper. TY and WW performed the experiments. TY, YH, JL, and XD analyzed the data. All authors contributed to the article and approved the submitted version.

## Funding

This work was funded by Southern University of Science and Technology (Y01226026), Key Laboratory of Molecular Design for Plant Cell Factory of Guangdong Higher Education Institute (2019KSYS006), the National Key R&D Program of China (grant 2017YFA0503800), and the National Natural Science Foundation of China (31621001).

## Conflict of Interest

The authors declare that the research was conducted in the absence of any commercial or financial relationships that could be construed as a potential conflict of interest.

## Publisher's Note

All claims expressed in this article are solely those of the authors and do not necessarily represent those of their affiliated organizations, or those of the publisher, the editors and the reviewers. Any product that may be evaluated in this article, or claim that may be made by its manufacturer, is not guaranteed or endorsed by the publisher.
