# Supplementary material for: SWELLMAP 2, a phyB-Interacting Splicing Factor, Negatively Regulates Seedling Photomorphogenesis in Arabidopsis
Source: Front Plant Sci. 2022 Feb 10;13:836519. doi: 10.3389/fpls.2022.836519 (PMC8867171; doi:10.3389/fpls.2022.836519)
Supplement: Supplementary file 1 [file Data_Sheet_1.docx]

Supplementary Material

**
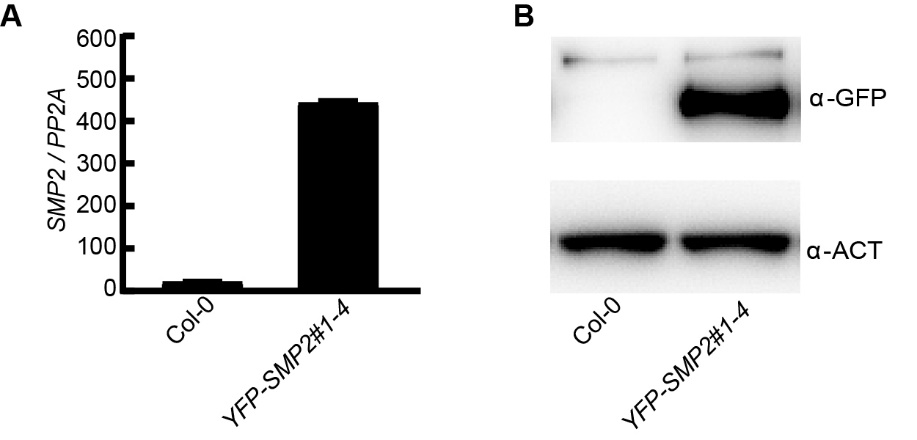
**

**Supplementary Figure 1.** Identification of *YFP-SMP2* transgenic plant. **(A)** *SMP2* expression level in Col-0 and *YFP-SMP2* plants. Total RNA was extracted from 5-day-old seedlings of Col-0, *YFP-SMP2* grown under constant white light condition. *PP2A* was used as the internal control. Error bars represent SD, n = 3. The experiments are performed three times with similar results. **(B)** Protein abundance of YFP-SMP2 in *YFP-SMP2* plants. Total proteins were extracted from 5-day-old seedlings of Col-0, *YFP-SMP2* grown under constant white light condition. YFP-SMP2 proteins were detected by immunoblot analysis with anti-GFP antibody. α-ACT, anti-Actin antibody, was used as a loading control.


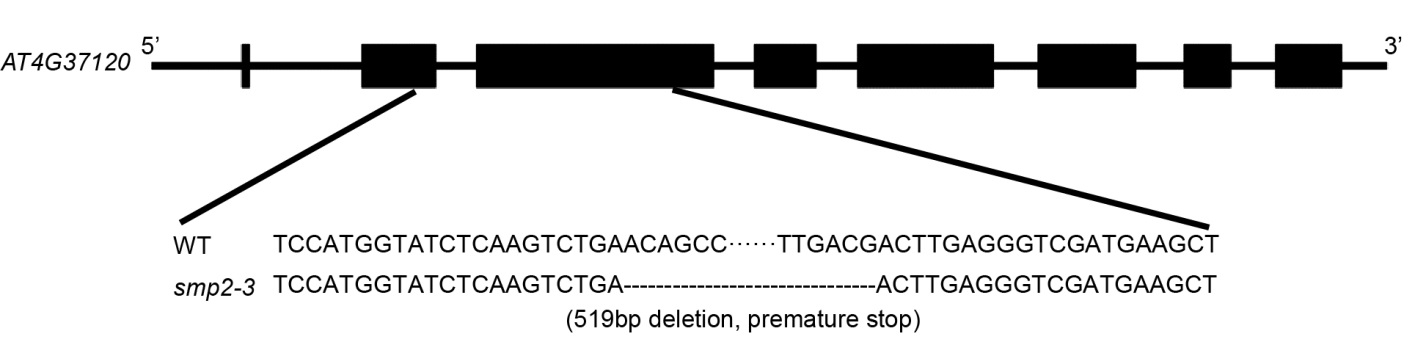


**Supplementary Figure 2.** The *smp2-3* mutant allele generated by CRISPR/Cas9. Black boxes in the gene body indicated the exon regions, horizontal lines indicated the intron regions.


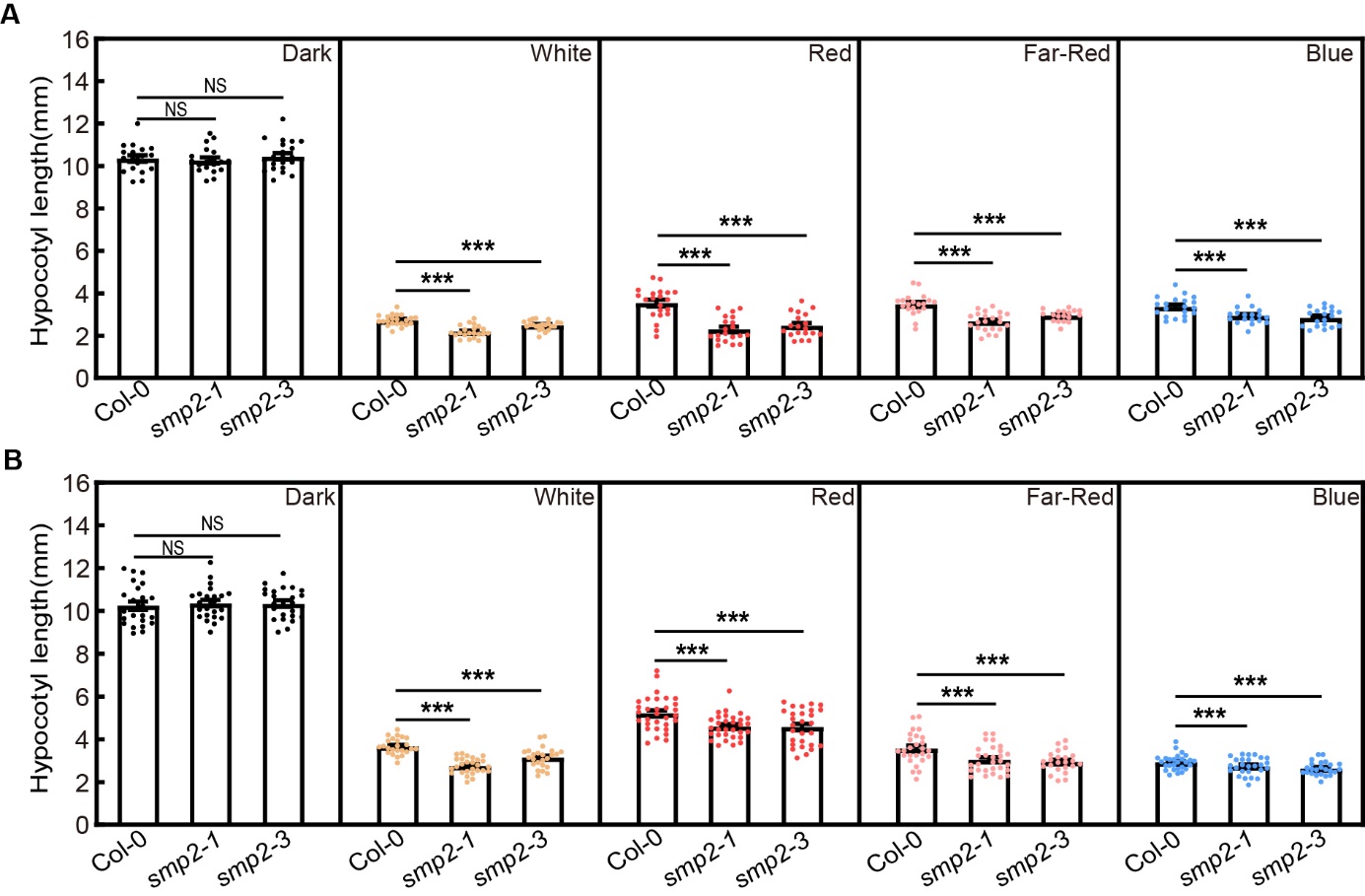


**Supplementary Figure 3.** Biological replicates of hypocotyl phenotypes of *smp2* mutants. Error bars represent standard deviation (SD), n ≥20; ***, *P*<0.001 (*t-test*); NS, not significant (*t-test*).

**
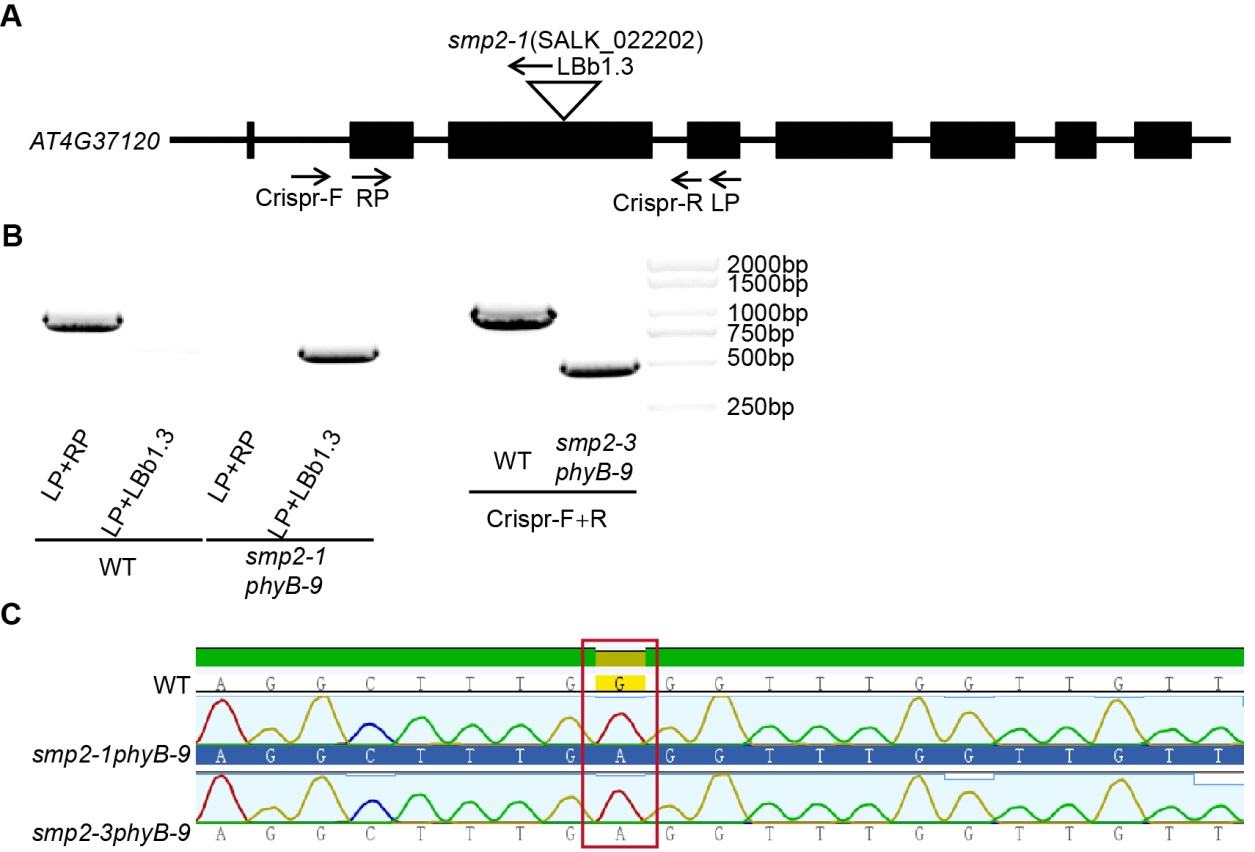
**

**Supplementary Figure 4.** Identification of homozygous *smp2-1 phyB-9* and *smp2-3 phyB-9* mutants. (A) The schematic diagram of primers used for genotyping *smp2-1* and *smp2-3*. (B) Identification of homozygous *smp2-1* and *smp2-3* by PCR with the indicated primers. (C) Identification of homozygous *phyB-9* allele by DNA sequencing. The red box indicated the G-to-A mutation in *phyB-9*.


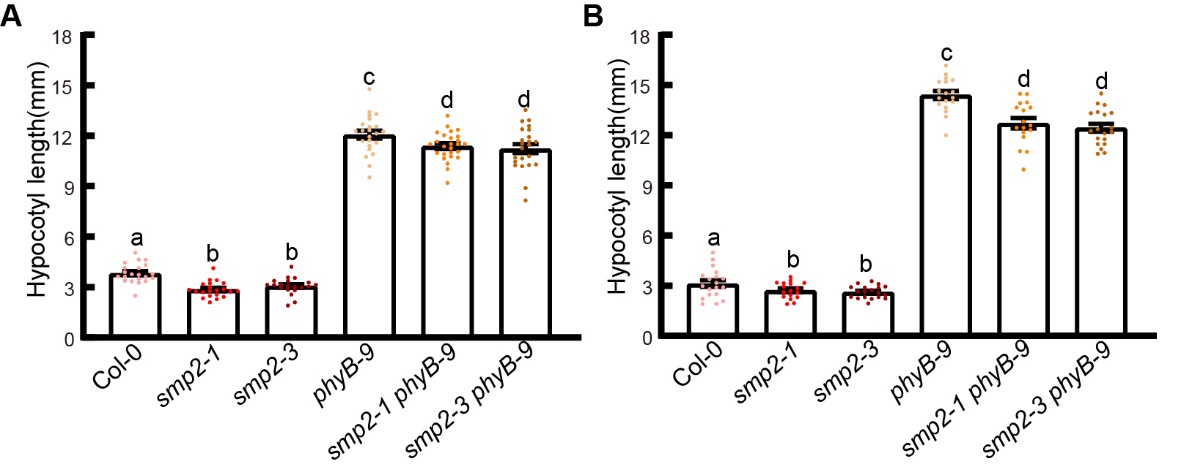


**Supplementary Figure 5.** Biological replicates of hypocotyl phenotypes of the mutants as indicated. Error bars represent SD, n ≥ 20. Letters above the bars indicate significant differences (*P* < 0.05), as determined by one-way analysis of variance (ANOVA) with Duncan’s post-hoc analysis.


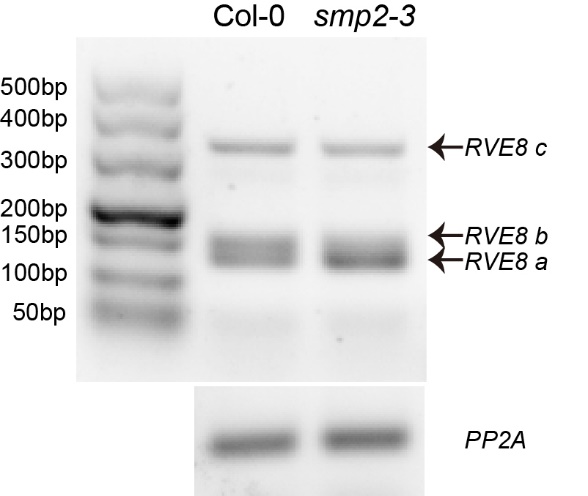


**Supplementary Figure 6.** *RVE8* splicing patterns in Col-0 and *smp2-3* by RT-PCR. Total RNA was extracted from Col-0 and *smp2-3* seedlings treated as described in **Figure 4A**. Sequences of related primers RVE8-RT-F1 and RVE8-PCR-R were listed in **Supplemental Table 1.**

**
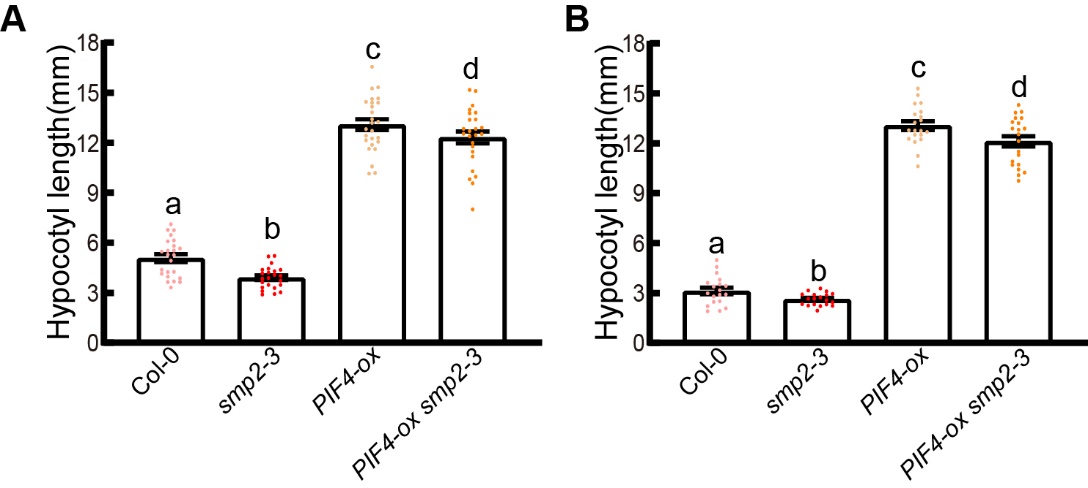
**

**Supplementary Figure 7.** Biological replicates of the hypocotyl phenotypes of the mutants as indicated. Error bars represent SD, n ≥ 20. Letters above bars indicate significant differences (*P* < 0.05), as determined by one-way analysis of variance (ANOVA) with Duncan’s post-hoc analysis.

**
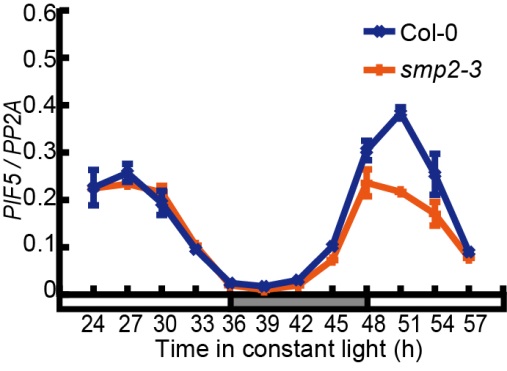
**

**Supplementary Figure 8.** Relative expression level of *PIF5* in Col-0 and *smp2-3* mutant. Seedlings were entrained in the diurnal cycles (12 h light / 12 h dark) for 6 days and then transferred to continuous white light (145 μmol·m^-2^·sec^-1^). Seedlings were harvested as described in **Figure 4A**. *PP2A* was used as an internal control. Error bars represent SD, n = 3.


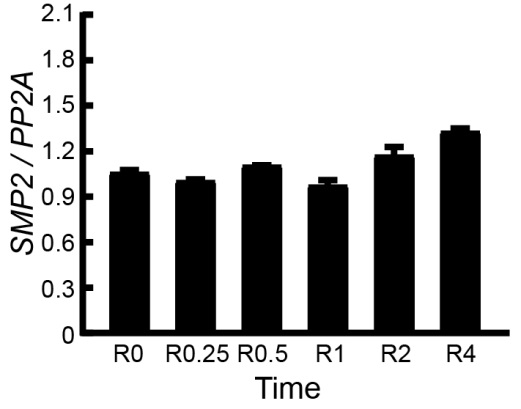


**Supplementary Figure 9.** *SMP2* expression is not regulated by red light. Seedlings were grown in the dark for 5 days and then transferred to red light (145 μmol·m^-2^·sec^-1^) for indicated times (hours). *PP2A* was used as the internal control. Error bars represent SD, n = 3. The experiments were performed three times with similar results.

**Supplemental Table 1.** Primers used in this study.

| **Primer name** | **Primer sequences (5’→3’)** |
| --- | --- |
| **Genotyping primers** | |
| *smp2-1* LP | CACGATGATCACAAACTAACCAAATAG |
| *smp2-1* RP | TCTGAACAGCCGGTATATGCGAA |
| *smp2-3* Crispr-F | TGCTATTGGCTAGATTCATTTTGG |
| *smp2-3* Crispr-R | GTCTTCTCTGATACGCAGATTCC |
| *phyB-9* seq-F | TGCTGTTCAATCGCAGAAAC |
| *phyB-9* seq-R | TCGCAGTGTGAGATCGAAAC |
| **Real-time qPCR** | |
| PP2A-RT-F | TATCGGATGACGATTCTTCGTGCAG |
| PP2A-RT-R | GCTTGGTCGACTATCGGAATGAGAG |
| SMP2-RT-F | TAAAGCTGGTCTCGCTCCTG |
| SMP2-RT-R | GCTTCAAACTCGGCTGTTCA |
| RVE8-RT-F1 | CCTCACAGTTAACTTATCAAACCCTG |
| RVE8-RT-F2 | GGATCCTATAAATTTCGAAACTGTTCT |
| RVE8 a-RT-R1 | TCATATGATAAGAGGACTTTCCTAGTGG |
| RVE8 b-RT-R2 | GCATCATTACAATCCGAAGTGGA |
| RVE8 c-RT-R3 | ACAATCCGACTAGCTGAGGT |
| RVE8-PCR-R | GCTGATTTGTCGCTTGTTGAGTTC |
| PRR5-RT-F | GCCGGGACAGAGTTCATACC |
| PRR5-RT-R | CTTGGGGAGAGTGAAGCAGG |
| TOC1-RT-F | AATAGTAATCCAGCGCAATTTTCTTC |
| TOC1-RT-R | CTTCAATCTACTTTTCTTCGGTGCT |
| ELF4-RT-F | CAAAGCAACGTTCTTCGACA |
| ELF4-RT-R | CGACAATCACCAATCGAGAA |
| GI-RT-F | ACTAGCAGTGGTCGACGGTTTATC |
| GI-RT-R | GCTGGTAGACGACACTTCAATAGATT |
| PIF4-RT-F | TGCATCACAACCGACCGTAA |
| PIF4-RT-R | AACTTCAGCTGCTCGACTCC |
| PIF1-RT-F | ACTGAATCCCGTAGCGAGGAAA |
| PIF1-RT-R | CTTGTTGCAGCGAGGTATAAGT |
| PIF3-RT-F | AGACAGGAACCCTTCTCCACC |
| PIF3-RT-R | GTGATGGCACTGACATAGGGA |
| PIF5-RT-F | GTGGAACAAGTGTAACCATTGACC |
| PIF5-RT-R | GATCCGATCTCTCCTCCTCCTT |
